# Kinetics of Hydroxyl Growth on Natural Rubber Depolymerization with H_2_O_2_/Fenton Using Infrared Spectroscopy

**DOI:** 10.3390/polym17131847

**Published:** 2025-07-01

**Authors:** Heri Budi Wibowo, Sutrisno Sutrisno, Hamonangan Rekso Diputro Sitompul, Retno Ardianingsih, Luthfia Hajar Abdilah, Kendra Hartaya, Ario Witjakso

**Affiliations:** 1 Research Centre for Rocket Technology, Research Organization of Aeronautics and Space, National Research and Innovation Agency, Bogor 16350, West Java, Indonesia; sutr004@brin.go.id (S.S.); hamo001@brin.go.id (H.R.D.S.); retn014@brin.go.id (R.A.); luth002@brin.go.id (L.H.A.); kend001@brin.go.id (K.H.); 2 Energy Conversion and Conservation Research Centre, Research Organization of Energy and Manufacture, National Research and Innovation Agency, Bogor 16340, West Java, Indonesia; ario001@brin.go.id

**Keywords:** hydroxylated natural rubber, Photo-Fenton, photochemical depolymerization, depolymerization kinetics, natural rubber

## Abstract

The kinetics of the depolymerization of natural rubber (NR) to hydroxyl-terminated natural rubber (HTNR) by hydrogen peroxide (H_2_O_2_) in the presence of a Fenton catalyst within an acidic milieu and under ultraviolet radiation has been rigorously examined utilizing infrared spectroscopy to determine the alterations in molar mass and the functional characteristics. The kinetic model was analyzed in accordance with the elementary reaction, encompassing the following mechanisms: the interaction between hydroxyl radicals and NR, producing radical NR and hydroxylated NR; the reaction wherein radical NR and hydroxyl radicals yield hydroxylated NR; and the subsequent reaction of hydroxylated NR with hydroxyl radicals producing lower radical NR, hydroxylated terminated NR, radical NR, and hydroxylated NR. The conversion of the NR polymer and the total hydroxyl content were discerned at the absorption bands of the CH_2_-CH_2_ and OH groups located at 850 cm^−1^ and 3400 cm^−1^, respectively. The absorption peak at 1850 cm^−1^ attributed to CH_3_ was employed as the reference group for calibration. The influence of the temperature on the depolymerization process conformed to the Arrhenius equation, characterized by activation energies of 750 K and 1200 K. The impact of the H_2_O_2_/Fenton ratio on the depolymerization process follows a power law with power coefficients of 1.97 and 1.82.

## 1. Introduction

Natural rubber has been extensively utilized across various applications owing to its sustainable and renewable characteristics. Innovative elastomeric materials can be synthesized through the modification of the structural composition of natural rubber. Natural rubber possesses the capability to undergo depolymerization, resulting in smaller natural rubber oligomers that feature hydroxyl or carboxyl functional groups at the terminal positions of their polymer chains. Novel materials derived from natural rubber have been produced via the polymer chain scission (depolymerization) of liquid natural rubber (LNR), yielding lower molar mass rubber oligomers, while incorporating reactive terminal groups to generate telechelic natural rubber (TLNR) [1,2,3]. One of the synthesized derivatives of natural rubber is hydroxyl-terminated natural rubber (HTNR), which possesses -OH functional groups at the terminal ends of the polymer and has been employed in various applications including grafting [4,5] and chain extension [6,7,8], as an adhesive [5,9,10,11], as a chain modifier [12,13,14,15], and as a binder in propellant formulations [16,17,18,19,20].

LNR depolymerization into HTNR has been executed through a variety of depolymerization reaction methodologies [21,22,23]. The synthesis of HTNR can be achieved via photochemical processes [24,25,26,27,28], metal catalysis [21,22,23,29,30,31], redox reactions [11,31], thermal degradation [32,33], and specific chemical oxidation techniques [27]. Ravindran [24,25] and Pham [26] investigated the application of radicals derived from H_2_O_2_ in a depolymerization reaction facilitated by ultraviolet irradiation from a mercury vapor lamp. In his initial experiment, Ravindran employed a 125 W UV mercury vapor lamp in conjunction with 30% H_2_O_2_ for a 30-h duration of LNR depolymerization [24], while in his subsequent trial, he utilized a 400 W mercury vapor lamp for a 52-h depolymerization reaction [25]. The outcomes of Ravindran’s first depolymerization experiment yielded a 90% HTNR product characterized by a M_n_ of 5000 g/mol; M_w_ of 8100 g/mol; a dispersity of 1.61; and a hydroxyl functionality of 1.91. His second endeavor also achieved a 90% HTNR product with a M_n_ of 4100 g/mol; M_w_ of 8300 g/mol; a dispersity of 2.02; and a marginally superior -OH functionality of 1.97. Pham [26] introduced the incorporation of FeSO_4_ as a Photo-Fenton catalyst to enhance the generation of hydroxyl radicals through the decomposition of H_2_O_2_. The utilization of a Photo-Fenton catalyst permitted Pham to operate with a lower specification of UV mercury vapor lamp compared to Ravindran’s setup. Pham employed a 160 W mercury vapor lamp with a radiation duration of 32 h for the depolymerization of LNR into HTNR, achieving a reported yield of HTNR products between 92 and 93% with -OH functionality ranging from 1.97 to 1.98 and a M_n_ of 3060. The depolymerization mechanisms elucidated in the studies conducted by Ravindran and Pham are consistent with a radical depolymerization paradigm, characterized by the preferential attack of hydroxyl radicals on the CH_2_-CH_2_ bond (Cα) at sites exhibiting lower steric effect.

Baharulrazi [16] and Giang [27] executed the depolymerization of LNR through a meticulously controlled reduction–oxidation methodology. Baharulrazi [16] employed Co-bis-acetylacetonate in conjunction with ethanol and NaBH_4_ to synthesize an oxidizing agent, NaBO_2_, which subsequently oxidized the CH_2_-CH_2_ bond (Cα), followed by a reduction utilizing NaBH_4_, yielding an HTNR product characterized by a M_n_ of 6691 g/mol, M_w_ of 27,560 g/mol, and a dispersity score of 4.12, all achieved within a reaction timeframe of one hour. Conversely, Giang [27] adopted a distinct methodology by utilizing a pH 9 borate buffer solution (comprising H_2_B_4_O_7_, NaCl, and Na_2_H_20_B_4_O_17_), THF, and 0.1 g of (NH_4_)_2_S_2_O_7_ to generate hydroxyl radical specimens via a redox reaction between the OH- ions and the borate buffer solution. The HTNR product obtained was isolated and demonstrated properties of M_n_ 4334 g/mol, M_w_ 11,702 g/mol, and a dispersity score of 2.7.

Azhar [34] implemented a synergistic photosensitizer system composed of methylene blue and rose Bengal in the degradation reaction of LNR under visible light exposure over a duration of fourteen days. The oxidation process transpired in two distinct phases: the initial phase involved the use of H_2_O_2_ and acetic acid in conjunction with a Na_2_WO_4_ catalyst. The primary oxidation reaction facilitated the transformation of LNR structures into their epoxidized variants, known as LENR, which subsequently underwent additional oxidation to yield hydroxylated LNR, denoted as LNR-OH. The hydroxyl functional groups present in LNR-OH, as established in Azhar’s investigation, were not solely located at the terminal ends but were also bonded to other carbon atoms (Cβ). A yield of 55.4% of LNR-OH products was obtained, exhibiting properties of M_n_ 1800 g/mol, M_w_ 3200 g/mol, and a dispersity score of 1.78.

The kinetics associated with the photochemical depolymerization of natural rubber or polyisoprene utilizing the H_2_O_2_/Fenton/Acid catalyst were scrutinized based on the radical reaction mechanism posited by Pham and Ravindran [25,26]. Hydroxyl radicals are produced through the redox reaction involving H_2_O_2_/Fenton/Acid in the presence of ultraviolet radiation. The depolymerization of rubber is initiated by the attack of hydroxyl radicals on the α-CH_2_-CH_2_ bond of natural rubber. The termination of depolymerization occurs due to radical–radical interactions. The ultimate product consists of hydroxyl-terminated natural rubber accompanied by byproducts that are lightly cross-linked with carbonyl, carboxyl, and hydroxyl functional groups. The characterization of molar mass and functionality is influenced by factors such as acidity, composition, UV radiation, and the catalyst employed. Variations in functional groups may serve as a foundational basis for the kinetics study pertaining to the depolymerization of natural rubber. The reaction rate is governed by the interactions between the polymer and the radicals. The stages of initiation and termination are characterized by rapid dynamics. It is postulated that the reaction may be relatively straightforward and adheres to a second-order reaction mechanism [25,35]. Nonetheless, this kinetics model may not adequately encapsulate the alterations in the molar mass and functionality of HTNR. However, the kinetics model premised on a molecular approach is proficient in describing the molar mass and functionality of hydroxyl-terminated polybutadiene [36] and the polycondensation of hydroxylated polybutadiene [37]. This kinetics model is anticipated to be applied to elucidate the depolymerization of natural rubber, particularly concerning the functionality and cross-linking characteristics. The radical polymer is categorized into mono-hydroxylated rubber (R-OH) and di-hydroxylated rubber (OH-R-OH). The incorporation of these functional groups serves to elucidate the properties and interlinkages of HTNR.

The kinetics of these processes have been examined through gravimetric analysis [25], viscometry techniques [25,26], and thermal methodologies [38,39]. Infrared spectroscopy represents the most straightforward and expedient approach for investigating the kinetics associated with polymerization and depolymerization [37]. Within this manuscript, the kinetics model was assessed utilizing infrared spectroscopy. The hydroxyl, carbonyl, carboxyl, and methyl functional groups were quantified through their respective infrared absorption spectra. The reference spectra employed pertain to the absorption characteristics of methyl (CH_3_).

## 2. Materials and Methods

### 2.1. Materials

Acetone, toluene, methanol, tetrahydrofuran (THF), sodium ascorbate, FeSO_4_·7H_2_O, H_2_O_2_ (50%, *w*/*w*), H_2_SO_4_, and NaOH were supplied by BRIN. Deproteinized liquid natural rubber (DPLNR) was supplied by the Indonesian Rubber Institute. Natural crumb rubber was obtained by coagulating DPLNR with acetone.

### 2.2. Methods

#### 2.2.1. Synthesis of HTNR

Natural crumb rubber (NCR) was masticated at a temperature of 30 °C for 60 min. Then, 15 g of the masticated NCR was dissolved in 150 mL toluene and charged into a 1000 mL glass reactor. The reactor was equipped with a magnetic stirrer, water heater, water condenser, and mercury vapor UV lamp 320 W. Precise amounts of Fenton reagent (H_2_O_2_ and Fe (II)) were added dropwise and continuously stirred with a magnetic bar. The mixture was homogenized with 50 mL THF. The reaction of the mixture was conducted at 60 °C and a pH of about 2.5–3.0 by the addition of H_2_SO_4_ in THF solution, and the molar ratio of H_2_O_2_/Fe (II) was set at 1.5 [26]. The UV radiation was exposed to the reactor after a 50 h reaction. The distance between the reactor wall and the UV beam source was 3 cm. Next, 0.06 g hydroquinone (about 0.02% *w*/*v* of the sample mixture) was dispersed to the solution and allowed to remain for a certain time. The water layer at the bottom was separated and removed, while the liquid rubber was recovered from the toluene layer by distilling the solvent at low pressure. The rubber product was washed using aquadest and methanol, respectively, and antioxidant sodium ascorbate 0.04 g (0.6 *w*/*w*) was added. The HTNR layer was separated and washed with toluene and methanol. The sample for the test and analysis was purified by the repeated precipitation of methanol from the toluene solution. The remaining solvents were removed by drying in a vacuum oven at 70 °C for 4 h.

#### 2.2.2. Analysis

FTIR (Fourier transform infrared) spectra of HTNR samples were tested using an FT-IR Spectrometer BRUKER Model: Alpha II by BRUKER OPTIK GmbH, Germany, for analysis, scanned from 400 to 4000 cm^−1^. The infrared spectra of HTNR were analyzed at 20 h intervals during a period of 80 h of UV radiation exposure. The H-NMR and C-NMR were used to measure the chemical shift, such as the H-shift and C-shift of the NR Crumb(t_0_) and HTNR samples (t_20_, t_40_, t_60_, t_80_). H-NMR and C-NMR analyses were carried out using NMR (nuclear magnetic resonance) BRUKER ASCEND 700 MHz with a 54 mm ASCEND Magnet by BRUKER GmbH, which was operated at 28 °C. The solvent used for H-NMR and C-NMR analysis was CDCl_3_ (deuterated chloroform).

The molar masses of the NR and HTNR were determined by GPC (gel permeating chromatography) Shimadzu LC-20AD by Shimadzu Corporation, Japan, with a DGU-20ASR degassing unit and an RID-10A Detector [40]. The GPC measurement used PEEK Columns of 8 μm, 50 mm × 7.5 mm, maintained at 30 °C, with THF as the mobile phase, flowing at 1.0 mL/min.

The hydroxyl, hydroperoxide, carbonyl, and carboxyl functional groups were quantitatively assessed employing attenuated total reflectance (ATR) methodologies, utilizing a precisely weighed sample of 0.3 g [41,42,43,44,45]. The concentration of the additive within the rubber matrix was subjected to thorough quantitative analysis via the attenuated total reflection Fourier transform infrared (ATR-FTIR) technique. By employing partial least squares (PLS) multivariate analysis, a significant prediction accuracy was achieved, yielding an error margin of approximately 0.15 wt% [46]. The broad absorption band associated with hydroxyl (O-H Stretching, b, 3600–3400 cm^−1^) alongside the C-O stretching absorption band characteristic of primary alcohols (m, 1310 cm^−1^) can be utilized to verify the existence of primary hydroxyl groups in hydrocarbon thermoplastic natural rubber (HTNR). Tetramethyl silane (TMS) was incorporated as a reference compound during the nuclear magnetic resonance (NMR) measurement. The variations in the 1H and 13C shielding shifts, along with the emergence of novel peaks, serve as indicators to distinguish between natural rubber (NR) and its depolymerized variant, HTNR.

#### 2.2.3. Kinetics Model

Ravindran (1988) and Pham (2015) proposed a mechanism of HTNR production from LNR depolymerization initiated by hydroxyl radical production from H_2_O_2_ decomposition under UV light radiation, with the presence of a Fenton catalyst or other catalyst in an acidic solution that undergoes a redox reaction [25,26].

(1) $$H_{2}O_{2}+\left[H^{+}\right]\overset{\;\;\;\;\;\;hʋ\;\;\;\;\;}{\to }\;{OH}^{*}+H_{2}O$$

(2) $$H_{2}O_{2}+{Fe}^{2+}+\left[H^{+}\right]\overset{\;\;\;\;\;\;hʋ\;\;\;\;\;}{\to }\;{OH}^{*}+{Fe}^{3+}+H_{2}O$$

The hydroxyl radical will carry out an attack on the CH_2_-CH_2_ bond with the Cα site that has a lower steric hindrance, which is the preferred attack point, to produce a hydroxylated natural rubber structure (AOH) and a radical natural rubber structure (A*), as depicted in Equation (3). The radical structure of natural rubber (A*) will react with another hydroxyl radical (OH*) to produce another hydroxylated natural rubber, which would have a lower molecular mass, as depicted in Equation (4). These reactions will occur steadily, resulting in a much smaller hydroxylated rubber structure size until the depolymerization reaction is terminated.
(3)
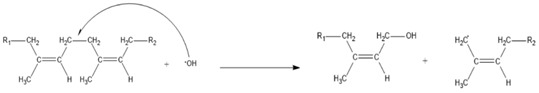

(4)
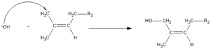



The reaction mechanism was not developed enough to illustrate the production of HTNR, which can be developed further by introducing the HTNR production reaction. The model of liquid deproteinated natural rubber (DPLNR) [*A*] reacted with OH radicals generated from the Fenton reaction. The hydroxyl radicals attack the Cα site (-CH_2_-CH_2_-), with the site of lower steric hindrance preferred, to produce a one-sided hydroxyl-terminated natural rubber structure [*AOH*] and a CH_2_ radical structure [A*], which will be terminated with OH radicals resulting another one-sided hydroxyl-terminated natural rubber structure [*AOH*].
(5)
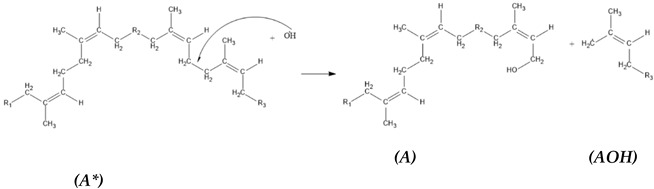

(6)
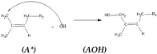

(7)
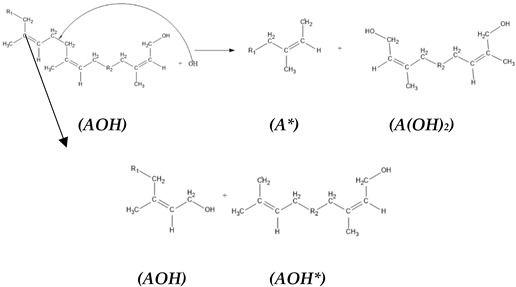



Structure [*AOH*], which has a one-sided hydroxyl group in termination, reacted further with hydroxyl radicals. We proposed two reaction pathways, which resulted in a one-sided hydroxyl-terminated natural rubber structure [F] similar to the previous reaction and a two-sided hydroxyl-terminated natural rubber structure [H]. The difference in reaction pathways is the radical formation [AOH*] and [A(OH)_2_], which may influence the characteristic of the two-sided hydroxyl-terminated natural rubber structure.
(8)
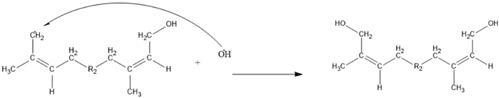



Hydroxyl radical (OH*) was produced by the decomposition of H_2_O_2_ under radiation of ultraviolet from a mercury lamp [24], sunlight [25], and catalyzation by a Fenton agent under ultraviolet radiation in an acidic solution [26]. The rate constant of hydroxyl radical production grew 1000–10,000 times in the presence of a Fenton catalyst [26]. The hydroxyl radical attack on natural rubber (A) resulted in a radical polymer structure (A*) and hydroxylated polymer (AOH) with a rate reaction constant of $k_{p}$. The radicalized polymer will react with hydroxyl radicals to produce a hydroxylated polymer at a rate reaction coefficient of $k_{t}$. The depolymerization reaction will occur continuously until the hydroxyl radical is depleted or a termination process occurs. The depolymerization equation of natural rubber followed Equations (9) and (10). The rate equation of depletion of the reacting polymer (*A*) will follow a second-order reaction and is presented in Equation (11), in which the concentration of the hydroxyl radical is excessive. [31].

(9) $$A+{OH}^{*}\overset{k_{p}}{\to }A^{*}+AOH$$

(10) $$A^{*}+{OH}^{*}\overset{k_{t}}{\to }AOH$$

(11) $$-\frac{d\left[A\right]}{dt}=k_{p}{\left[A\right]}^{2}$$

The formation rate of polymer radicals [A*] is steady against time, in which we assume the concentration is constant $\left(d\left[A^{*}\right]/dt=0\right)$. The rate of the radical polymer [A*] formation is presented in Equation (12), while the rate of the hydroxylated natural rubber [*AOH*] is depicted in Equation (13).

(12) $$\frac{d\left[A^{*}\right]}{dt}=k_{p}{\left[A\right]}^{2}-k_{t}\left[A^{*}\right]=0$$

(13) $$\frac{d\left[AOH\right]}{dt}=k_{p}{\left[A\right]}^{2}+k_{t}\left[A^{*}\right]=k_{p}{\left[A\right]}^{2}+k_{p}{\left[A\right]}^{2}=2k_{p}{\left[A\right]}^{2}$$

Referring to Equation (13), the kinetics of the depolymerization reaction of natural rubber are sufficiently represented by Equation (11). The differential Equation (11), with natural rubber’s initial concentration of [*A*]_o_, with time from 0 to t, can be solved as Equation (14). The value of the reaction rate coefficient $k_{p}$ can be obtained by using the data from the concentration of natural rubber [*A*] against time.

(14) $$-\frac{1}{{\left[A\right]}_{0}}+\frac{1}{\left[A\right]}=k_{p}t$$

The equation reaction in (14) cannot properly explain the changes in hydroxyl functionality during the depolymerization of NR polymer to HTNR. The equation of the reaction can be solved by presenting the formation phase of hydroxylated terminated natural rubber from NR polymer, which will be written as Equations (15)–(19). In the initial phase, natural rubber reacts with the hydroxyl radical to form a polymer radical [A*] and a hydroxylated polymer [*AOH*] with a reaction rate coefficient of $k_{1}$. The polymer radical will react further with another hydroxyl radical to form a new hydroxylated polymer [*AOH*] with a rate constant of $k_{2}$. The hydroxylated polymer will react with the hydroxyl radical to form a hydroxylated terminated natural rubber [*A*(*OH*)_2_] and another polymer radical with a reaction rate coefficient of $k_{3}$. The hydroxylated polymer could also react with a hydroxyl radical to form a hydroxylated polymer radical [AOH*] and hydroxylated polymer of [*AOH*] with the rate constant of $k_{4}$. The radical of the hydroxylated polymer [*AOH*] will undergo a termination reaction with a hydroxyl radical to form a hydroxylated terminated natural rubber [*A*(*OH*)_2_] with a rate constant of $k_{5}$.

(15) $$A+{OH}^{*}\overset{k_{1}}{\to }A^{*}+AOH$$

(16) $$A^{*}+{OH}^{*}\overset{k_{2}}{\to }AOH$$

(17) $$AOH+{OH}^{*}\overset{k_{3}}{\to }A^{*}+{A(OH)}_{2}$$

(18) $$AOH+{OH}^{*}\overset{k_{4}}{\to }{AOH}^{*}+AOH$$

(19) $${AOH}^{*}+{OH}^{*}\overset{k_{5}}{\to }{A(OH)}_{2}$$

The reaction of the hydroxyl radical with reactant [*A*] is a second-order reaction with a rate equation similar to Equations (13) and (14), with the rate constant being $k_{1}$. The equation of the reactant depletion rate is depicted in Equation (21).

(20) $$-\frac{d\left[A\right]}{dt}=k_{1}{\left[A\right]}^{2}$$

(21) $$-\frac{1}{{\left[A\right]}_{0}}+\frac{1}{\left[A\right]}=k_{1}t$$

The polymer radical [A*] concentration is relatively constant during the depolymerization. Thus, the change in the rate of the polymer radical formation is equal to zero. The concentration of the polymer radical is derived in Equation (22). The concentration of the hydroxylated polymer radical [*AOH] is also relatively constant, with the change in the rate of the hydroxylated polymer radical formation being zero, as presented in Equation (24).

(22) $$+\frac{d\left[A^{*}\right]}{dt}=k_{1}{\left[A\right]}^{2}+k_{3}\left[AOH\right]-k_{2}\left[A^{*}\right]=0$$

(23) $$\left[A^{*}\right]=\frac{k_{1}{\left[A\right]}^{2}+k_{3}\left[AOH\right]}{k_{2}}$$

(24) dAOH*dt=k4AOH*−k5AOH=0

(25) AOH*=k5k4AOH

The change in the growth of concentration of the hydroxylated polymer (AOH) is represented in Equation (26). Substituting Equation (26) into Equations (25) and (24) results in Equation (27). The value of the [*AOH*] concentration is a function of the concentration of [*A*] with a rate constant of $k_{1}$.

(26) $$\frac{d\left[AOH\right]}{dt}=k_{1}{\left[A\right]}^{2}+\left(k_{3}+k_{4}\right)\left[AOH\right]-k_{4}\left[AOH\right]+k_{2}\left[A^{*}\right]$$

(27) $$\frac{d\left[AOH\right]}{dt}={2k}_{1}{\left[A\right]}^{2}$$

By combining Equation (27) with Equation (21), we have Equation (28), with the solving of the differential Equation (28) under the initial condition as boundary [*A*]_0_, [*AOH*]_0_, and a reaction time of 0 to t resulting in Equation (29).

(28) $$\frac{d\left[AOH\right]}{d[A]}=2$$

(29) $$\left[AOH\right]-{\left[AOH\right]}_{0}=2\left[A\right]-{2\left[A\right]}_{0}o$$

The change in the rate of formation of the hydroxylated terminated polymer is presented in Equation (30). The incorporation of Equations (25) and (26) into Equation (30) results in Equation (31) with $k_{34}=k_{3}+k_{4}$. The substitution of Equation (25) into Equation (30) results in the equation of [*A*(*OH*)_2_] as a function of [*A*]. The solution to the differential equation of (31) by the initial conditions as boundary [*A*]_0_, [*AOH*]_0_, and [*A*(*OH*)_2_]_0_ from time 0 to t results in Equation (33).

(30) d[AOH2]dt=k3AOH+k5AOH.=k3AOH+k4AOH=k34AOH

(31) $$\frac{d[A{\left(OH\right)}_{2}]}{d[A]}=\left(\frac{k_{34}}{k_{1}}\right)\frac{{\left[AOH\right]}_{0}+2[A]}{\left[A\right]}$$

(32) $$\left[A{\left(OH\right)}_{2}\right]-{\left[A{\left(OH\right)}_{2}\right]}_{0}=\left(\frac{k_{34}}{k_{1}}\right)\left({\left[AOH\right]}_{0}ln\frac{\left[A\right]}{{\left[A\right]}_{0}}+2\left(\left[A\right]-{\left[A\right]}_{0}\right)\right)$$

(33) $$\left[AOH\right]+\left[A{\left(OH\right)}_{2}\right]={\left[A{\left(OH\right)}_{2}\right]}_{0}+{\left[AOH\right]}_{0}+2\left[A\right]+\left(\frac{k_{34}}{k_{1}}\right)\left({\left[AOH\right]}_{0}ln\frac{\left[A\right]}{{\left[A\right]}_{0}}+2\left(\left[A\right]-{\left[A\right]}_{0}\right)\right)$$

According to equation (25), the value of the reaction rate coefficient $k_{1}$ is obtained from the slope of 1/[*A*] − 1/[*A*]_0_ plotted against the reaction time (t). If the concentration of the hydroxylated polymer of [*AOH*] and [*A*(*OH*)_2_] is measured at any given time, then the value of (*k*_34_/*k*_1_) is obtained from the relationship of $\left[AOH\right]+\left[A{\left(OH\right)}_{2}\right]$ plotted against ${\left[AOH\right]}_{0}ln\frac{\left[A\right]}{{\left[A\right]}_{0}}+2\left(\left[A\right]-{\left[A\right]}_{0}\right)$. The value of $k_{34}$ can be determined from the calculated value of $k_{1}$ and $\left(k_{34}/k_{1}\right)$.

The equation of reaction can be simplified into Equations (13), (15) and (34) to solve the reaction kinetics.

(34) $$AOH+{OH}^{*}\overset{k_{34}}{\to }A^{*}+{A(OH)}_{2}+{AOH}^{*}+AOH$$

The hydroxyl functionality value can be determined by using the value of the hydroxylated polymer [*AOH*], which had functionality of 1, and [*A*(*OH*)_2_], with a functionality score of 2 as depicted in Equation (35).

(35) $$f_{OH}=\frac{\left[AOH\right]+2[A{\left(OH\right)}_{2}]}{\left[AOH\right]+[A{\left(OH\right)}_{2}]}$$

The average molecule weight of polyurethane can be calculated by using a Stockmayer equation [36,47]. The molar mass average of a number of polymer’s depolymerization can be calculated using Equation (36), which is the mole fraction of the initial polymer *n_A_*, the weighted average of the polymer *M_A_*, the functionality *f_A_*, and the converted fraction *p_A_*.

(36) $${\bar{M}}_{n}=\frac{n_{A}M_{A}}{\left(n_{A})-(n_{A}f_{A}p_{A}\right)}$$

(37) $${\bar{M}}_{w}=\frac{{n_{A}}^{2}M_{A}}{\left({n_{A}}^{2})-({{n_{A}}^{2}{f_{A}}^{2}p_{A}}^{2}\right)}$$

The influence of the reaction temperature against the reaction rate coefficient was studied by observing the reaction temperature variation toward the changes in reaction rates [23,48]. The relationship of the reaction temperature with the reaction rates coefficients of $k_{1}$ and $k_{34}$ followed the Arrhenius Equations (38) and (39) with *A*_1_ and *A*_34_ as the frequency factors, *Ea*_1_ and *Ea*_34_ as the activation energies, R as the ideal gas constant, and T as the reaction temperature. By plotting the value of ln(*k*) against (−1/*T*), the activation energy values of *Ea*_1_/*R* and *Ea*_34_/*R* can be derived.

(38) $$k_{1}=A_{1}exp\left(\frac{-{Ea}_{1}}{RT}\right)$$

(39) $$k_{34}=A_{34}exp(\frac{-{Ea}_{34}}{RT})$$

## 3. Results and Discussion

### 3.1. HTNR Characterization

**Figure 1:**
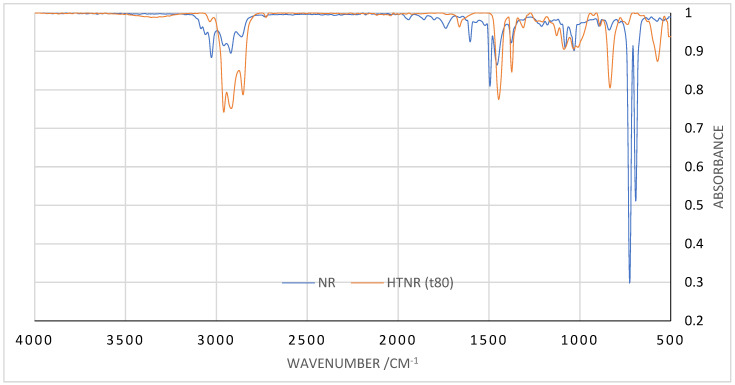
NR and HTNR infrared spectra under specific reaction conditions.

Figure 1 presents the infrared spectra of the HTNR product and NR under a reaction temperature of 30 °C and a pH system of 2. The concentration of [H_2_O_2_] was 2 mol L^−1^, the ratio of [H_2_O_2_]/[Fe^2+^] was set at 1.5, and the ratio of [A]/[H_2_O_2_] was equal to 5. The samples were irradiated under UV lights of 320 Watts for 80 h of radiation time (t_80_).

The following presents the absorption on IR spectra: both HTNR and NR show absorption in 3040–3032 cm^−1^ (m), 2980–2958 cm^−1^ (s), 2862 cm^−1^ (s), 2726 cm^−1^ (s) (C-H str.); 1661 cm^−1^ (m, C=C, cis-vinylene); 1446 cm^−1^ (s), 1377 cm^−1^ (s) (C-H def.); 891 cm^−1^ (m, -CH_3_ def.); 830 cm^−1^ (s, C-H out of plane def. in –CHR=CCR1) as the major IR absorption band characteristics for cis-1,4-polyisoprene (cis-1,4-PIP) [17,24,26]. The HTNR product is characterized by a broad absorption band at 3600–3400 cm^−1^, characteristic of OH stretching vibration, and an absorption band at 1310 cm^−1^ (m, C-O str., aliphatic primary alcohol). Meanwhile, the presence of other groups on the LNR chains prepared in neutral and alkaline media can be attributed to the appearance of carbonyl peaks (C=O) at 1720 cm^−1^ for ketone and 1739 cm^−1^ for aldehyde, respectively. The chain breaking of NR at the carbon–carbon single bond is usually due to an attack of radicals and leaving a hydroxyl end group. In contrast, an oxidizing agent can oxidize the broken carbon–carbon double bond in the NR chains to leave a carbonyl end group [31,48].

**Figure 2:**
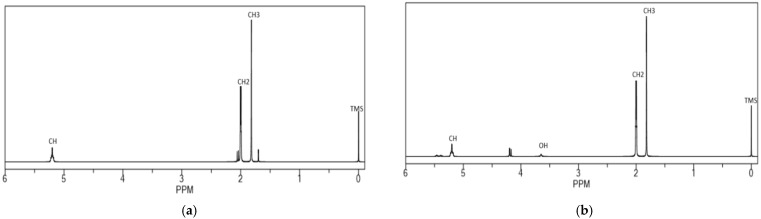
H-NMR spectra of (**a**) NR and (**b**) HTNR, which were prepared using the photo-Fenton process.

**Figure 3:**
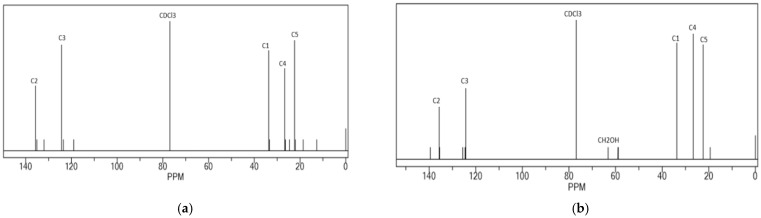
C-NMR spectra of (**a**) NR and (**b**) HTNR, which were prepared using the photo-Fenton process.

The ^1^H-NMR and ^13^C-NMR spectra of HTNR, acquired following a radiation duration of 20 h at a temperature of 30 °C in CDCl_3_ with [H_2_O_2_] set at 2 mol L^−1^, a [H_2_O_2_]/[Fe^2+^] ratio of 1.5, and an [A]/[H_2_O_2_] ratio of 5, along with a pH of 2 and irradiation from a 320 Watt UV lamp, are illustrated in Figure 2 and Figure 3. The ^1^H-NMR spectra exhibited absorption peaks at δ = 1.679 p.p.m. [s; -CH_3_ (5), 3H]; δ = 2.042 p.p.m. [brs; -CH_2_-(1) and -CH_2_-(4), 4H]; δ = 5.125 p.p.m. [m; CH (3), 1H]. Additionally, minor peaks emerged in the range of δ = 1.254–1.611 ppm, suggesting the existence of byproducts. The minor peaks at δ = 2.69 ppm further clarify that an epoxy group formation transpired [25,26]. The signal attributable to hydroxyl protons from hydroxymethyl indicates the nonexistence of functional groups within the chemical shift range of δ = 4.0 ppm to 4.2 ppm.

The ^13^C-NMR spectra delineated the presence of cis-1,4-PIP at the following chemical shifts: C1: δ = 32.23 p.p.m.; C2: δ = 135.16 p.p.m.; C3: δ = 125.03 p.p.m.; C4: δ = 26.38 p.p.m.; C5: δ = 23.4 p.p.m. Minor peaks were noted at δ = 78.39, 76.98, and 75.57 p.p.m., attributable to CDCl3, as well as δ = 60.847 and 64.540 p.p.m. This observation may be ascribed to α-carbons bonded to hydroxyl groups in molecular structures analogous to (I) and (II) [25,26]. The protons associated with allylic hydroxyl groups in the ^1^H-NMR spectra were obscured by the multiplet observed at δ = 5.125 p.p.m., corresponding to the >C=C-H protons (Equation (9)). Numerous minor peaks were also discernible in the ^13^C-NMR spectrum of HTNR (Figure 2b) within the δ range of 2.00 to 3.53 p.p.m., indicating the presence of side products due to epoxy group formation. The existence of the hydroxyl group from CH_2_-OH was corroborated by the detection of absorption peaks at δ = 60.847 p.p.m. and 64.540 p.p.m., characteristic of the α-carbons of allyl-alcohol in the ^13^C-NMR spectrum of HTNR, suggesting the presence of terminal hydroxyl groups in the resultant product. The allylic hydroxyl protons in the ^1^H-NMR spectra were obscured by the multiplet at δ = 5.125 p.p.m., corresponding to the >C=C-H protons. All other characteristic signals for both NR and HTNR were noted in the ^1^H-NMR spectrum (Figure 2a,b): δ = 5.08 p.p.m., (=CH); δ = 2.00 p.p.m., (-CH2-); δ = 1.67 p.p.m., (-CH3), in addition to the 13C-NMR spectrum (Figure 3): δ = 135.012 p.p.m., (C2 atom); δ = 124.900 p.p.m., (C3 atom); δ = 32.216 p.p.m., (C1 atom); δ = 26.409 p.p.m., (C4 atom); and δ = 23.433 p.p.m., (C5 atom). Further evidence was the fact that there was no observed change in the absorption band of the isoprene unit, i.e., at 836 cm^−1^ in the FTIR spectrum.

### 3.2. The Role of Infrared Absorption in Depolymerization Analysis

The kinetics of the depolymerization reaction were studied by determining the specific absorption peaks of HTNR and NR’s functional groups. The formation of the hydroxyl group could be characterized by an absorption peak of O-H stretch at a wave number of 3400 cm^−1^. The NR Polymer was undergoing degradation by the breaking of the CH_2_-CH_2_ bond, which can be indicated by the decline of the sharp absorption peak at 830 cm^−1^. The peak at wavenumber 1610 cm^−1^ had a lot of interferences to be used as a reference, while the absorption peak of the methyl CH_3_ group could be used as the reference with the absorption peaks at 2980 cm^−1^, which is relatively unchanged. As a matter of reaction kinetics, the absorption peak at 830 cm^−1^ could be used to observe the reduction of the reactant, while the absorption peak of 3400 cm^−1^ can be used to determine the quantity of hydroxyl group at any given time. Reactant conversion to the product was calculated by Equation (40)

(40) $$x[A]=\frac{A_{830}-A_{2980}}{A_{2980}}$$

**Figure 4:**
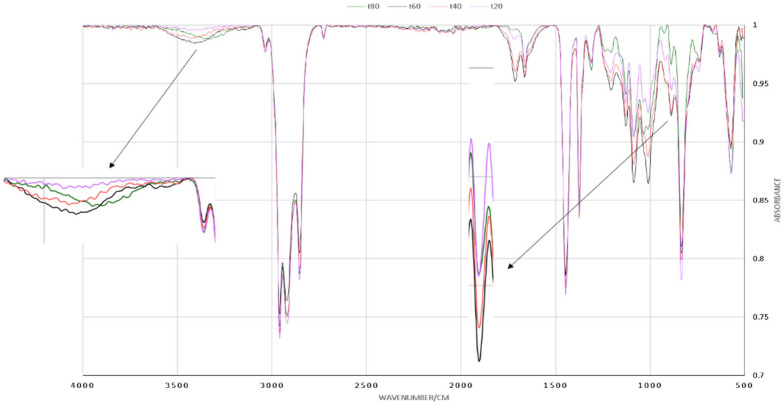
The concentration decline in CH_2_-CH_2_ and the growth in CH_2_-OH concentration at wave numbers 830 cm^−1^ and 3400 cm^−1^ from NR depolymerization by H_2_O_2_/Fenton.

The observed changes at wavenumbers 830 cm^−1^, 2980 cm^−1^, and 3400 cm^−1^ for every 20 h of observation for the depolymerization of NR with hydrogen peroxide with [H_2_O_2_] = 2 mol L^−1^, a ratio of [H_2_O_2_]/[Fe^2+^] = 1.5, and a ratio of [*A*]/[H_2_O_2_] = 5, pH = 2, irradiated by 320 Watts of UV light for 80 h (t_80_) are shown in Figure 4. There was an indication of the significant growth of hydroxyl concentration [OH] at wave number 2400 cm^−1^ and a notable concentration decline in reactant [*A*], with the reduction in the methylene group absorption at 830 cm^−1^. The quantity of the methyl group remains constant at a wavenumber of 2980 cm^−1^, which can be used as a reference absorption peak.

### 3.3. The Kinetics of HTNR Formation Reaction

**Figure 5:**
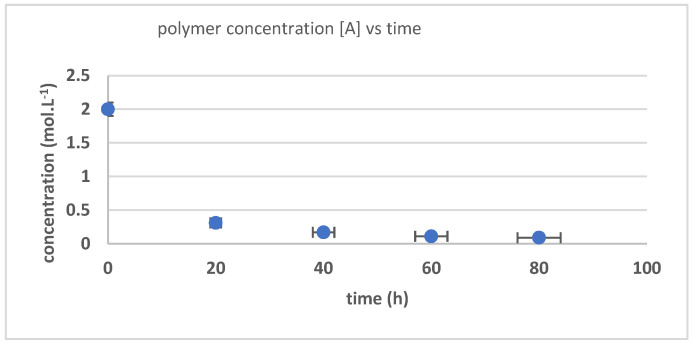
The changes in [A] concentration against HNTR reaction time.

Figure 5 presents the concentration changes of polymer [A] against time. The polymer concentration declined as the reaction time increased. After 60 h of depolymerization reaction, the concentration decline was relatively small with a more than 90% conversion rate. The reaction was conditioned under excessive hydroxyl radical conditions; thus, the reaction rate coefficient of hydroxyl group formation was not studied.

**Figure 6:**
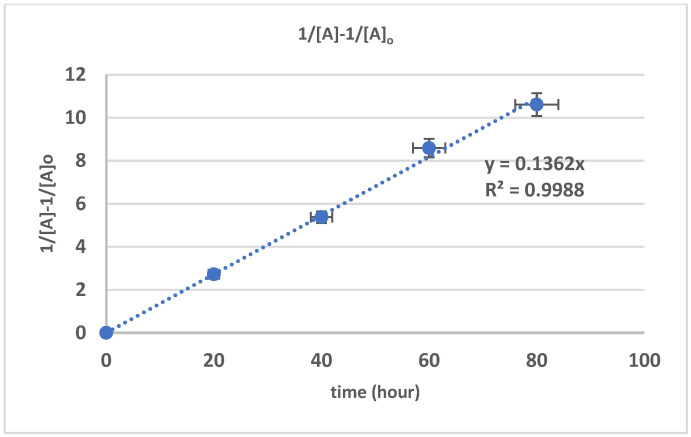
The relationship of 1/[A] − 1/[A]_0_ against time.

The conversion reaction of the polymer [A] followed a second-order reaction, which is depicted by the graph of 1/[A] − 1/[A]_0_ against time as a linear line. The second-order depolymerization reaction with hydroxyl radical is in accordance with the previous work on the depolymerization reaction of natural rubber with hydrogen peroxide under high temperature [48], UV radiation [24], and the catalyst addition of NaNO_2_ [31]. The relationship of 1/[A] − 1/[A]_0_ against time following Equations (3)–(13) is shown in Figure 6. The value of $k_{1}$ was obtained as the slope of that graph, with the calculated value of $k_{1}$ being 0.01356 L·mol^−1^·s^−1^ with R^2^ = 0.9988.

**Figure 7:**
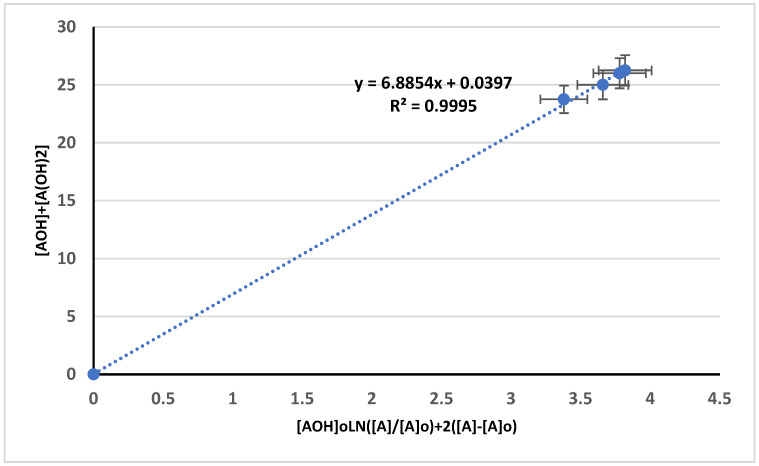
Relationship between [AOH] + [A(OH)_2_] and [AOH]_0_Ln([A]/[A]_o_) + 2([A] − [A]_o_).

The ratio of the degradation reaction rate coefficient of AOH against A $(k_{34}/k_{1})$ was obtained as the slope of the concentration of the measured hydroxyl group [AOH] + [A(OH)_2_] graph against [AOH]_0_Ln([A]/[A]_0_) + 2([A] − [A]_0_), as illustrated in Figure 7, which followed Equations (3)–(24), with a regression coefficient R^2^ = 0.9995. From the calculated value of $(k_{34}/k_{1})$, the value of $k_{34}$ was measured at 0.9337 mol·L^−1^·s^−1^. The value of $k_{34}>k_{1}$ indicates that most of the AOH reacted with the hydroxyl radical to form hydroxyl-terminated natural rubber (HTNR). Thus, the dominant product is the HTNR.

**Figure 8:**
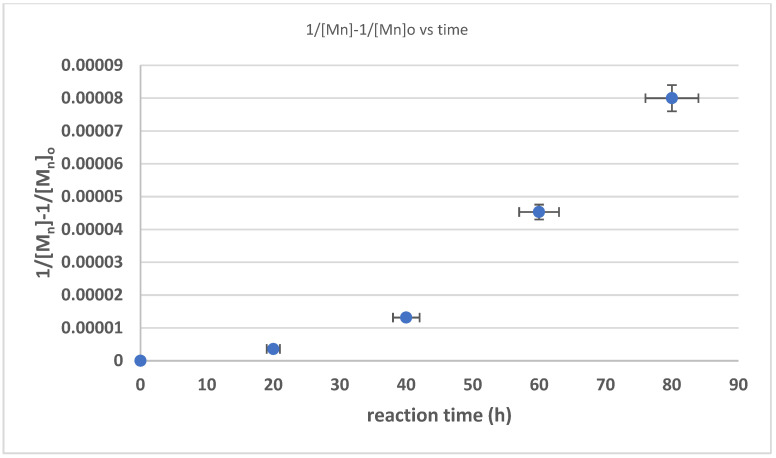
Relationship between 1/[M_n_] − 1/[M_n_]_0_ and reaction time.

The changes in the average molar mass (M_n_) and weight average molar mass (M_w_) from the measurement and simulation using Equations (3)–(27) are presented in Figure 8. The results of the measurement and simulation showed a similar trend. The error in the estimation rate was determined to be 2.7%. The result showed a similar trend to the previous work of Ravindran [25], Wisetkhamsai [31], and Pham [26], in which the decline in molar mass is not linear. The decline with the second-order base reaction revealed a decline in 1/[M_n_] − 1/[M_n_]_0_ against time, which was also not linear (linearity score of 0.820). These results strengthen the hypothesis that the reaction formed hydroxyl-terminated natural rubber.

**Figure 9:**
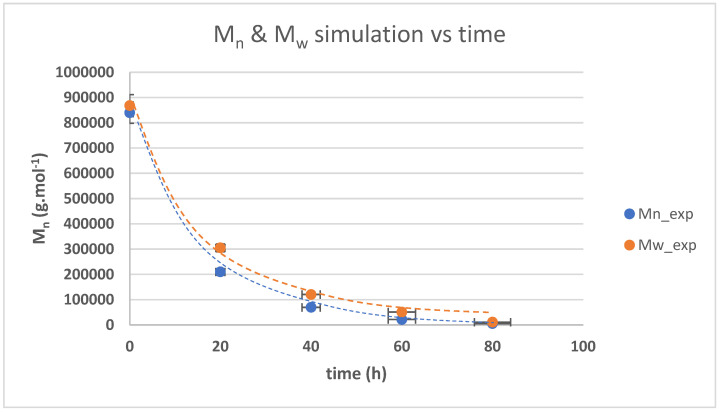
Relationship between M_n_ and M_w_ against time and their simulation results.

The simulation results of the weight, average molar mass, average molar mass at any given time, and the measurement results are presented in Figure 9. The simulation result showed a slightly lower value than the experiment result. The error in the estimation rate was determined to be 0.7% for M_n_ and 0.2% for M_w_. The trend was similar to the previous results of Pham [26] and Ravindran [25]. The value of the dispersity score (polydispersity index) from this work is around 2, as presented in Table 1. The dispersity score rose steadily against time until the reaction time of 60 h, then dropped slightly in the next 20 h of reaction time.

**Figure 10:**
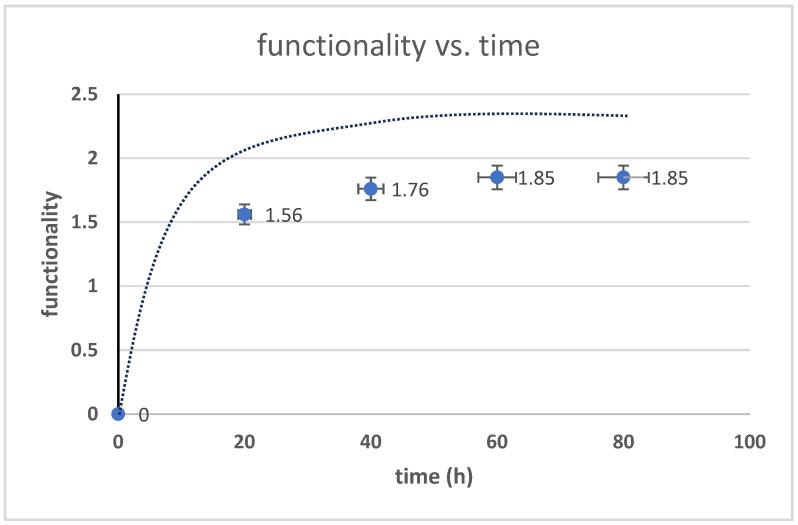
Hydroxyl functionality changes over time.

The functionality of HTNR was calculated using Equations (3)–(27), while the relationship between the simulation and real-time measurement is depicted in Figure 10. The calculated value was lower than the simulated value, with an error rate of 2%. The functionality value was well-matched by the kinetics model.

### 3.4. The Influence of Reaction Temperature

**Figure 11:**
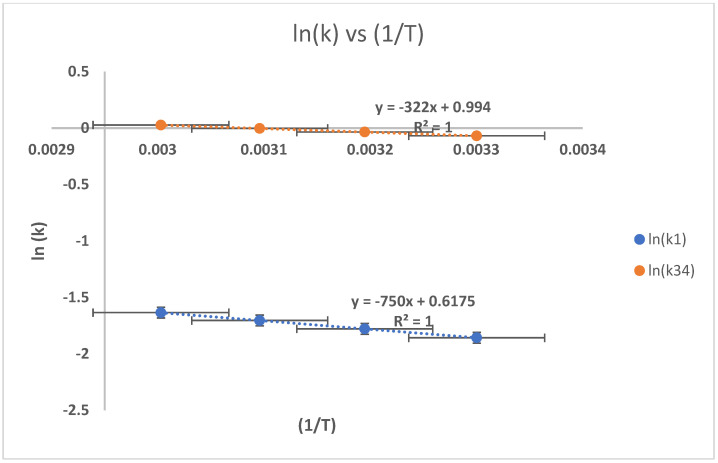
Relationship of ln (k) against (1/T).

The influence of the reaction time against the reaction rate of HTNR formation was studied under variations in the reaction temperature of 30, 40, 50, and 60 °C, with other variables fixed at [H_2_O_2_] = 0.2 mol L^−1^, [H_2_O_2_]/[Fe(II)^++^] = 1.5, pH = 2.5, and [H_2_O_2_]/[A] = 1.5. The value of $k_{1}$ and $k_{34}$ were obtained from the graph at 1/[M_n_] − 1/[M_n_]_0_ against the reaction time and [AOH] + [A(OH)_2_] against [AOH]_0_ Ln([A]/[A]_0_) + 2([A] − [A]_0_). The calculated values of $k_{1}$ and $k_{34}$ were used to model the relationship of ln k against (–1/T), as depicted in Figure 11. The activation energy value (Ea_1_/R dan Ea_34_/R) was determined as the slope of that graph, which followed the Arrhenius Equations (3) to (28) and (3) to (29). The values of the pre-exponential factor of A_1_ and A_34_ are reported as intercepts of the graph. The calculated values of Ea_1_/R, Ea_34_/R, A_1_, and A_34_ are 750 K, 1200 K, 1.854, and 2.72. The Ea/R positive value indicates that the reaction is exothermic. The value coefficient of determination (R^2^) is 1 for k_1_ and 1 for k_34_. The value of Ea_1_/R < Ea_34_/R revealed that the formation reaction of radicals is faster than the cleavage reaction of AOH. According to Pham [26], the reactivity of the hydroxyl radical in attacking the site of the CH_2_-CH_2_ bond is much higher compared to the CH_2_-CH_2_ bond, in which the hydroxyl group is already present due to more positive electronegativity. Thus, the reaction is quite selective. The growth of the $k_{1}$ and $k_{34}$ values for every 10 degrees of temperature increase revealed those are lower than twice the initial $k_{1}$ and $k_{34}$ values, indicating that the depolymerization reaction is a diffuse regime reaction. Diffusivity is the step that controls the reaction, and the reaction is considered to be quite quick. The Ea value is calculated as -slope *R; the ${Ea}_{1}$ is 90 J, while the ${Ea}_{34}$ is 144 J.

### 3.5. The Influence of Reactant and Catalyst Ratio

**Figure 12:**
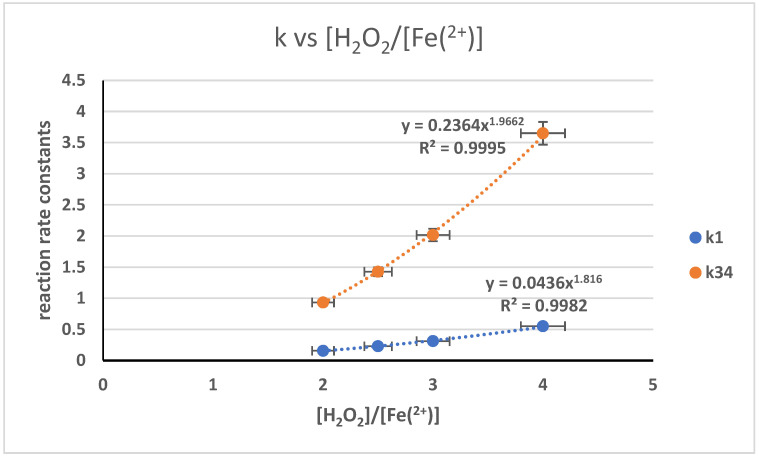
Reaction rate coefficient relationship toward the changes in reactant/catalyst concentration.

The optimal ratio of [A]/[H_2_O_2_] optimal was 1.5, which indicates the excessive presence of hydroxyl radicals. Meanwhile, the influence of the [H_2_O_2_]/[Fe(II)] ratio against the reaction rate was studied using the ratio variations of 1, 1.5, 2.0, and 2.5. The ratio influence of [A]/[H_2_O_2_] toward the calculated reaction rate coefficient is depicted in Figure 12. The graph illustrates the relationship of $k_{1}$ and $k_{34}$ against [A]/[H_2_O_2_] as a power equation, with a power value of 1.97 against $k_{1}$ and 1.82 toward $k_{34}$. The coefficient of determination is 0.9995. This result is in accordance with previous works [31] of similar depolymerization reactions using NaNO_2_ catalysts, which depicts the influence of the concentration of H_2_O_2_ and NaNO_2_ against radical production as a power equation.

## 4. Conclusions

In general, the depolymerization reaction kinetics of NR’s conversion to HTNR using H_2_O_2_ under the presence of a Fenton catalyst in an acidic environment and ultraviolet radiation can be studied using infrared spectroscopy to examine the changes in the molecular mass and the functionality of the HTNR product. HTNR synthesis has the following reaction mechanism: (a) hydrogen peroxide reacts with Fenton in the acid condition to produce the hydroxyl radical, (b) the hydroxyl radical is reacted with NR to produce radical NR and hydroxylated NR, (c) the radical NR is reacted with the hydroxyl radical to produce hydroxylated NR, and (d) the hydroxylated NR is reacted with the hydroxyl radical to produce lower radical NR, hydroxylated terminated NR, radical NR, and hydroxylated NR.

The analysis of the product revealed the presence of HTNR with hydroxyl group absorption at a wave number of 3400 cm^−1^. Depolymerization also occurred with a decline in the average molar mass for each period of sample measurement. The NR polymer decline due to conversion to HTNR was observed at the absorption band of the CH_2_-CH_2_ group at 850 cm^−1^, the value of total hydroxyl produced at 3400 cm^−1^, and the absorption of the CH_3_ reference group, which remained unchanged at 1850 cm^−1^.

The reaction kinetics were studied by employing an assumption of excessive hydroxyl concentration. The NR polymer degradation reaction follows a reaction order of 2 with a rate constant $k_{1}$. The hydroxylated natural rubber production reaction conformed to a first-order reaction with a rate constant of $k_{34}$. The number average of molar mass (M_n_), weight average of molar mass (M_w_), and OH functionality matched well. The reaction rate coefficient data of $k_{34}>k_{1}$ described the conversion reaction of HTNR as quickly occurring. The average functionality is between 1.8 and 1.9, revealing a dominant HTNR production. The reaction reached an optimum rate at 60 h, at which the OH functionality was at its maximum value. Based on the constructed kinetic model and supporting experimental results, the kinetic parameters of the depolymerization reaction are elementary reactions, similar to other depolymerization reactions.

The influence of reaction temperature against the reaction rate coefficient followed the Arrhenius reaction, with activation energy values of ${Ea}_{1}/R$ and ${Ea}_{34}/R$ of 750 K and 1200 K, respectively. Meanwhile, the effect of the concentration of H_2_O_2_/Fenton followed the Fenton reaction, with a power coefficient of 1.97 against $k_{1}$ and 1.82 toward $k_{34}$.

## Figures and Tables

**Table 1:** Dispersity of HTNR.

Time (h)	M_n__exp/g/mol	M_w__exp/g/mol	1/[M_n_} − 1/[M_n_]_0_	Dispersity
0	840,000	868,000	0	1.033333
20	210,000	305,100	3.57 × 10^−6^	1.452857
40	69,700	120,500	1.32 × 10^−5^	1.728838
60	21,500	51,000	4.53 × 10^−5^	2.372093
80	5300	11,100	8.00 × 10^−5^	2.09434

## Data Availability

The data presented in this study are available upon request from the corresponding authors.
